# KRAS Mutations Predict Response and Outcome in Advanced Lung Adenocarcinoma Patients Receiving First-Line Bevacizumab and Platinum-Based Chemotherapy

**DOI:** 10.3390/cancers11101514

**Published:** 2019-10-09

**Authors:** Áron Kristof Ghimessy, Áron Gellert, Erzsebet Schlegl, Balazs Hegedus, Erzsebet Raso, Tamas Barbai, Jozsef Timar, Gyula Ostoros, Zsolt Megyesfalvi, Balazs Gieszer, Judit Moldvay, Ferenc Renyi-Vamos, Zoltan Lohinai, Mir Alireza Hoda, Thomas Klikovits, Walter Klepetko, Viktoria Laszlo, Balazs Dome

**Affiliations:** 1 Department of Thoracic Surgery, National Institute of Oncology-Semmelweis University, 1122 Budapest, Hungary; aronghimessy@gmail.com (Á.K.G.); werte02@gmail.com (Á.G.); megyesfalvi_2007@yahoo.com (Z.M.); dr.gieszer@gmail.com (B.G.); drrenyivamosferenc@gmail.com (F.R.-V.); 2 Department of Tumor Biology, National Koranyi Institute of Pulmonology–Semmelweis University, 1122 Budapest, Hungary; erschlegl@gmail.com (E.S.); drmoldvay@hotmail.com (J.M.); lohinaiz@gmail.com (Z.L.); 3 Department of Thoracic Surgery, Ruhrlandklinik, University Duisburg-Essen, 45239 Essen, Germany; Balazs.Hegedues@rlk.uk-essen.de; 4 2nd Department of Pathology, Semmelweis University, 1091 Budapest, Hungary; raso.erzsebet@med.semmelweis-univ.hu (E.R.); tbarbai@gmail.com (T.B.); jtimar@gmail.com (J.T.); 5 Tumor Progression Research Group, Hungarian Academy of Sciences-Semmelweis University, 1091 Budapest, Hungary; 6 8th Department of Pulmonology, National Koranyi Institute of Pulmonology, 1122 Budapest, Hungary; drostorosgyula@gmail.com; 7 MTA-SE NAP, Brain Metastasis Research Group, Hungarian Academy of Sciences, 1091 Budapest, Hungary; 8 Division of Thoracic Surgery, Department of Surgery, Comprehensive Cancer Centre Vienna, Medical University Vienna, A-1090 Vienna, Austria; mir.hoda@meduniwien.ac.at (M.A.H.); thomas.klikovits@meduniwien.ac.at (T.K.); walter.klepetko@meduniwien.ac.at (W.K.)

**Keywords:** bevacizumab, non-small-cell lung cancer, advanced-stage lung adenocarcinoma, platinum-based chemotherapy, KRAS mutation

## Abstract

Bevacizumab, combined with platinum-based chemotherapy, has been widely used in the treatment of advanced-stage lung adenocarcinoma (LADC). Although KRAS (V-Ki-ras2 Kirsten rat sarcoma viral oncogene homolog) mutation is the most common genetic alteration in human LADC and its role in promoting angiogenesis has been well established, its prognostic and predictive role in the above setting remains unclear. The association between KRAS exon 2 mutational status and clinicopathological variables including progression-free survival and overall survival (PFS and OS, respectively) was retrospectively analyzed in 501 Caucasian stage IIIB-IV LADC patients receiving first-line platinum-based chemotherapy (CHT) with or without bevacizumab (BEV). EGFR (epidermal growth factor receptor)-mutant cases were excluded. Of 247 BEV/CHT and 254 CHT patients, 95 (38.5%) and 75 (29.5%) had mutations in KRAS, respectively. KRAS mutation was associated with smoking (*p* = 0.008) and female gender (*p* = 0.002) in the BEV/CHT group. We found no difference in OS between patients with KRAS-mutant versus KRAS wild-type tumors in the CHT-alone group (*p* = 0.6771). Notably, patients with KRAS-mutant tumors demonstrated significantly shorter PFS (*p* = 0.0255) and OS (*p* = 0.0186) in response to BEV/CHT compared to KRAS wild-type patients. KRAS mutation was an independent predictor of shorter PFS (hazard ratio, 0.597; *p* = 0.011) and OS (hazard ratio, 0.645; *p* = 0.012) in the BEV/CHT group. G12D KRAS-mutant patients receiving BEV/CHT showed significantly shorter PFS (3.7 months versus 8.27 months in the G12/13x group; *p* = 0.0032) and OS (7.2 months versus 16.1 months in the G12/13x group; *p* = 0.0144). In this single-center, retrospective study, KRAS-mutant LADC patients receiving BEV/CHT treatment exhibited inferior PFS and OS compared to those with KRAS wild-type advanced LADC. G12D mutations may define a subset of KRAS-mutant LADC patients unsuitable for antiangiogenic therapy with BEV.

## 1. Introduction

The KRAS (V-Ki-ras2 Kirsten rat sarcoma viral oncogene homolog) protein, encoded by the KRAS proto-oncogene, is a small GTPase (guanosine triphosphatase) that plays a key role in regulating various cell functions [1]. Alterations of the *KRAS* gene are typically missense mutations that can lead to the oncogenic conversion of KRAS resulting in the constitutive activation of its effector pathways and thus cancer development and progression [2]. KRAS is frequently mutated in pancreatic and colorectal cancer (CRC), and in lung adenocarcinoma (LADC). With an incidence of up to 30%, KRAS mutation is the most common driver mutation in LADC. The most prevalent G12C and G12V KRAS mutation subtypes are associated with smoking, while the G12D subtype has been observed in those who have never smoked [3,4]. Several other rare mutations of KRAS codon 12, 13, and 61 have also been reported [3].

The prognostic and predictive power of the KRAS mutation in non-small-cell lung cancer (NSCLC) patients remains controversial. It was first reported in the late 1980s that KRAS mutation is associated with poorer survival [5,6], and since then several groups confirmed these findings [7,8]. However, most of these studies were rather heterogeneous in terms of histology, tumor stage, and methodologies of KRAS mutation detection. Although two different meta-analyses concluded that KRAS mutation is a negative prognosticator in LADC [9,10], the most comprehensive study of more than 1500 NSCLC patients (including 300 KRAS-mutant cases) from four trials of adjuvant chemotherapy (CHT) reported that KRAS mutation had no clear prognostic or predictive relevance with regards to response to CHT [11].

Previously, our group performed a mutation subtype-specific analysis of 505 stage III–IV LADC patients treated with platinum-based CHT and found that there were no significant differences in progression-free survival (PFS) and overall survival (OS) among patients with wild-type (WT), codon 12, and codon 13 KRAS mutations. Importantly, however, G12V KRAS-mutant patients tended to have a higher response rate and a modestly longer median PFS [12].

The importance of subtype-specific KRAS mutation analysis was further highlighted in the preclinical study of Garassino et al. These authors investigated the role of different KRAS mutation subtypes (G12C, G12V, and G12D) in the in vitro chemosensitivity of human NSCLC cells and found that the expression of G12C was associated with a reduced response to cisplatin and an increased sensitivity to taxol and pemetrexed. In the same study, G12D mutation led to resistance to taxol and sensitivity to sorafenib, whereas the G12V mutation sensitized the cells to cisplatin [13].

Increased expression and the negative prognostic role of vascular endothelial growth factor (VEGF, the key angiogenic cytokine) have been reported in most solid tumors including NSCLC [14,15]. Several phase 2 and 3 clinical trials demonstrated that the addition of bevacizumab (BEV, a humanized monoclonal antibody against the VEGF-A isoform) to CHT improves the PFS and OS of NSCLC patients [16,17,18,19,20]. Accordingly, BEV in combination with platinum-based CHT was approved for the first-line treatment of patients with advanced-stage NSCLC by the FDA (U.S. Food and Drug Administration) and the EMA (European Medicines Agency) in 2006 and 2007, respectively. The efficacy of BEV in a real-life setting in Hungary was shown in the Avalanche study [21].

Although the RAS/RAF/MEK/ERK signaling pathway has been implicated in the regulation of VEGF expression and angiogenesis [22], only a few studies have investigated the effect of KRAS mutation on the efficacy of BEV therapy. Most studies focused on CRC, where the addition of BEV to CHT prolonged survival regardless of KRAS mutational status [23,24,25,26]. Two different groups, however, demonstrated that G12V, G12A [27], and G12D [28] KRAS mutations are associated with poor outcome in metastatic CRC patients receiving BEV. As for nonsquamous NSCLC, in a phase 2 trial evaluating the addition of neoadjuvant BEV to CHT, Chaft et al. found that no patient (0 out of 10) with KRAS mutation showed a pathological response to neoadjuvant BEV/CHT, in comparison to 11 of 31 KRAS WT patients [29]. In another small study of stage IV NSCLC, BEV therapy was associated with improved OS and PFS in KRAS WT (*n* = 26), but not in KRAS-mutant (*n* = 16) patients [30]. Here, we report the results of the first study, to our knowledge, of amino acid substitution-specific KRAS mutational status analysis in a large cohort of BEV/CHT-treated stage III–IV Caucasian patients.

## 2. Results

### 2.1. Incidence of KRAS Mutations in LADC Patients Treated with Bevacizumab and Chemotherapy

All patients had advanced LADC and Caucasian background. Patients with tumors harboring an EGFR mutation were excluded. One hundred and seventy patients of the full cohort of 501 cases were identified as KRAS-mutant (33.9%) and 331 (66.1%) as KRAS WT (see Table 1A,B). While 38.5% (*n* = 95) of the patients treated in the BEV/CHT group were KRAS-mutant (Table 1A), in the CHT group (Table 1B) this ratio was 29.5% (*n* = 75) (*p* = 0.012). There were no significant differences between the BEV/CHT and CHT groups with respect to age (*p* = 0.193), smoking status (*p* = 0.072), gender (*p* = 0.506), and tumor stage (*p* = 0.610) (data not shown). The only difference was seen in performance status (PS): there were more ECOG (Eastern Cooperative Oncology Group) 0 (vs. EVOG 1) patients in the BEV/CHT group than in the CHT-alone group (*p* = 0.031; data not shown), which might be due to the BEV selection criteria. In the BEV/CHT subcohort, 35 (36.8%), 19 (20%), and 20 (21%) cases were classified as G12C, G12D, and G12V mutants, respectively (Table S1). Other rare (i.e., *n* < 3) KRAS exon 2 mutation subtypes (G12A, G12R, G12S, G13C and G13D) were also found in the BEV group. Subtype-specific mutations were technically not assessable in 21 cases (Table S1).

In order to study the clinical relevance of KRAS mutations, we performed comparative statistical analyses of KRAS status and clinicopathological variables in both the BEV/CHT (Table 1A) and the CHT subcohorts (Table 1B). As for the BEV/CHT group, ever-smoking and KRAS mutational statuses showed a significant positive association (*p* = 0.008; see Table 1A). KRAS mutation was also significantly more common in female BEV/CHT patients (vs. males; *p* = 0.002; see Table 1A). ECOG status and clinical stage did not differ significantly between KRAS-mutant and KRAS WT patients in the BEV/CHT group (*p* = 0.056 and *p* = 0.16, respectively; see Table 1A). The presence of KRAS mutation was not associated with age in the BEV/CHT group (*p* = 0.09; see Table 1A). Of note, we did not detect significant associations of KRAS mutational status with age, smoking status, gender, ECOG status, stage, or OS in the CHT group (Table 1B). While the reasons for the differences in the associations between KRAS mutational status and clinicopathological variables in the BEV/CHT vs. the CHT subcohorts are not entirely clear, a possible explanation is that they are due to the selection criteria for BEV therapy.

### 2.2. The Presence of KRAS Mutations has Clinical Utility in Predicting Disease Outcome in LADC Patients Receiving Concurrent Antiangiogenic and Chemotherapy

**Figure 1:**
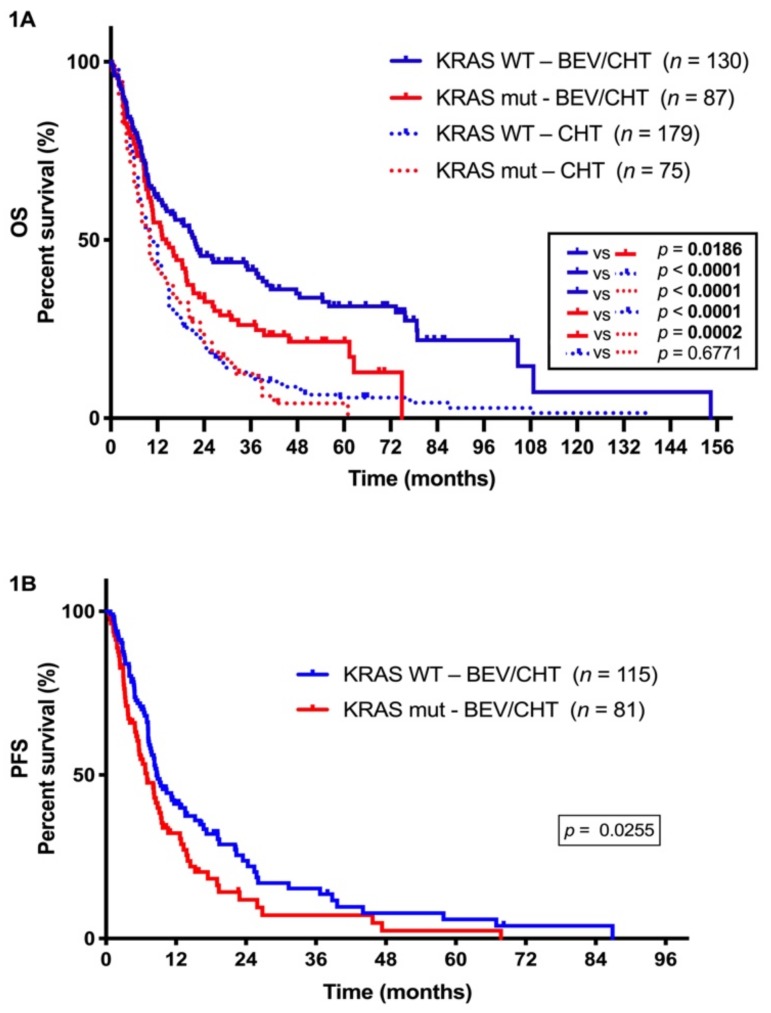
Kaplan-Meier plots for the overall survival (OS) (**A**) and progression-free survival (PFS) (**B**) in lung adenocarcinoma (LADC) patients according to V-Ki-ras2 Kirsten rat sarcoma viral oncogene homolog (KRAS) mutation status. (**A**) LADC patients with KRAS wild-type (WT) tumors and receiving bevacizumab/chemotherapy (BEV/CHT) had significantly increased median OS (vs. those with KRAS WT tumors and receiving CHT only; median OS 21.57 vs. 14.23 months, respectively, *p* = 0.0186, log-rank test). Median OS was also increased in KRAS-mutant LADC patients receiving BEV/CHT compared to those treated with CHT only (median OSs were 18 vs. 10 months, respectively, *p* = 0.0002, log-rank test). No significant differences in OS have been observed for patients receiving CHT only and with KRAS WT versus KRAS-mutant tumors (median OSs were 11 vs. 10 months, respectively *p* = 0.6771, log-rank test). Of note, in the BEV/CHT group, patients with KRAS WT LADC had a significantly better OS than those with tumors harboring KRAS mutations (median OSs were 39 vs. 18 months, respectively, *p* = 0.0186, log-rank test). (**B**) Similarly, in the BEV/CHT group, patients with KRAS WT LADC had significantly longer median PFS (vs. those with KRAS-mutant tumors; median PFSs were 8.63 vs. 7.03 months, respectively, *p* = 0.0255, log-rank test).

As expected, patients in the BEV/CHT group had significantly longer median OS than those receiving CHT only (*p* < 0.0001, log-rank test; Figure S2). This difference was even more remarkable when only KRAS WT patients were compared (*p* < 0.0001, log-rank test; see Figure 1A). Notably, the addition of BEV to CHT was also associated with a significant benefit in OS if KRAS-mutant patients were compared with those in the CHT-alone subcohort (*p* = 0.0002, log-rank test; see Figure 1A).

We next investigated whether the KRAS mutational status influences the efficacy of CHT with or without BEV in advanced LADC. There was no difference in OS between patients with KRAS-mutant versus KRAS WT tumors in the CHT-alone group (*p* = 0.6771, log-rank test; see Figure 1A). Importantly, however, in the BEV/CHT group we found that KRAS-mutant LADC patients had a significantly shorter median PFS and OS than did KRAS WT patients (*p* = 0.0255 and *p* = 0.0186, respectively, log-rank test; see Figure 1A,B). In support of this, multivariate Cox regression analyses revealed that KRAS status (mutant vs. WT) at diagnosis influenced OS (hazard ratio (HR) 0.645, 95% confidence interval (CI) 0.458–0.908, *p* =  0.012) and PFS (HR 0.597, 95% CI 0.402–0.887, *p* =  0.011) independently from age (continuous; *P* values were 0.081 and 0.628, respectively), gender (female vs. male; *p* values were 0.005 and 0.001, respectively), smoking status (never vs. ever; *p* values were 0.907 and 0.835, respectively), ECOG PS (0 vs. 1; *P* values were 0.193 and 0.177, respectively) and tumor stage (III. vs. IV; *p* values were 0.048 and 0.617, respectively; see Table 2). These analyses also identified more advanced tumor stage as a significant independent negative prognostic factor for OS, but not for PFS (*p* values were 0.048 and 0.617, respectively; see Table 2). Gender proved to be an independent prognosticator for both OS and PFS in a multivariate Cox regression model as well (*p* values were 0.005 and 0.001, respectively; see Table 2).

### 2.3. Distinct Efficacy of BEV/CHT in Advanced LADC Patients with Different Subtype-Specific KRAS Mutations

**Figure 2:**
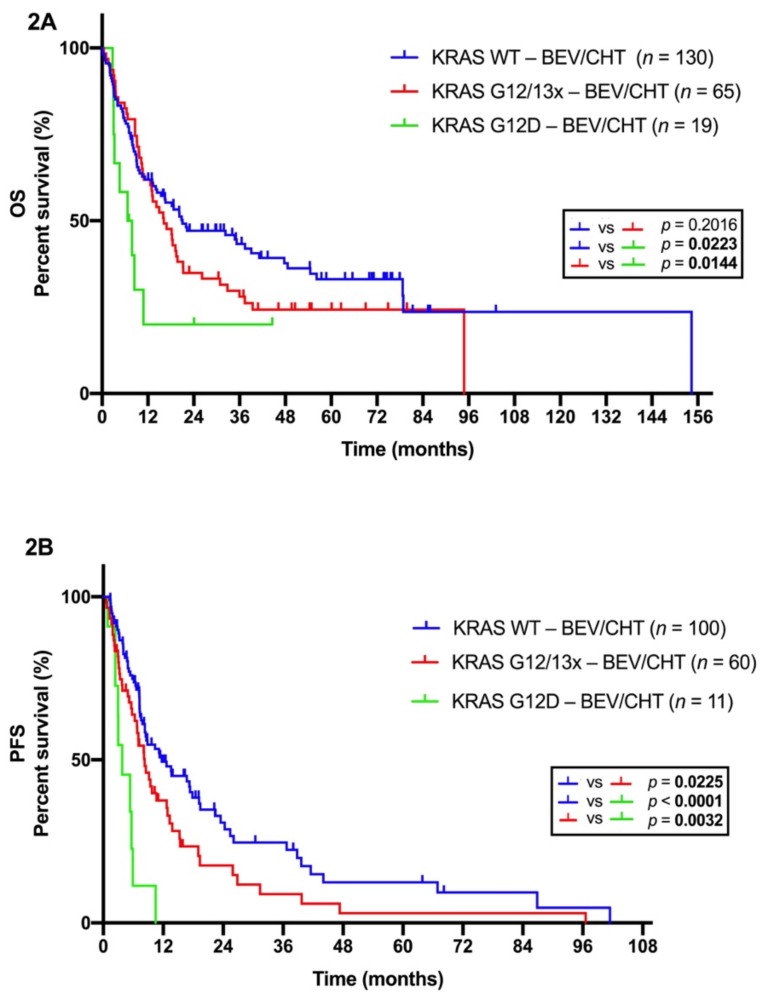
Kaplan-Meier plots for the OS (**A**) and PFS (**B**) in LADC patients receiving BEV/CHT according to subtype-specific codon 12 KRAS mutations. (**A**) KRAS G12D mutation was associated with significantly shorter OS in LADC patients (vs. KRAS G12x and 13x mutations or WT KRAS; median OSs were 7.2, 16.1, and 21 months, respectively, *p* values were 0.0144 and 0.0223, respectively, log-rank test). (**B**) LADC patients with tumors harboring KRAS G12D mutations had also significantly shorter median PFS than those with other codon 12 (G12x) and 13 (G13x) KRAS-mutant or with KRAS WT tumors (median PFSs were 3.7, 8.27, and 11.7 months, respectively; *p* values were 0.0032 and <0.0001, respectively; log-rank test).

Next, we looked at the clinicopathological characteristics of KRAS codon 12-mutant LADC patients receiving BEV/CHT and performed a statistical analysis on their associations with amino acid-specific mutational status. We identified 35 (36.8%) G12C, 19 G12D (20%), 20 G12V (21%), three G12A (3.2%%), one G12S (1%), one G12R (1%), three G13D (3.1%), and one G13C (1%) cases. Significant associations of subtype-specific KRAS mutational status with age, smoking status, gender, ECOG PS, or tumor stage were not detected (Table S1). Importantly, patients with KRAS G12D mutant tumors had a significantly shorter OS than those presenting with KRAS WT or with other KRAS codon 12 or 13 mutant (G12/13x) tumors (*p* = 0.0223 and *p* = 0.0144, respectively; log-rank test; see Figure 2A). In line with the OS data, KRAS G12D mutation conferred a significant disadvantage for PFS compared with KRAS WT (*p* < 0.0001; log-rank test; see Figure 2B) or all the other codon 12 or 13 KRAS (G12/13x) mutations (*p* = 0.0032; log-rank test; see Figure 2B). Of note, the OS of G12D KRAS-mutant patients in the BEV/CHT group was comparable to that of patients in the CHT-alone subcohort (Figure S3).

## 3. Discussion

Although KRAS is the most frequently mutated oncogene in NSCLC, our knowledge on the effect of KRAS mutation on the response to BEV in lung cancer is very limited. Biomarkers of BEV efficacy including imaging markers and circulating levels of angiogenic cytokines have been tested in both preclinical and clinical studies. For instance, VEGF levels in immunodepleted plasma of cancer patients were found to be significantly reduced following BEV treatment [31]. However, VEGF-A, as measured using an Enzyme-linked immunosorbent assay that recognizes all VEGF-A isoforms, was not predictive in a comprehensive evaluation of four phase III trials of BEV in CRC, NSCLC, and renal cancer [32]. Interestingly, recent data suggest use of TP53 (tumor protein 53) status as a biomarker for the response to BEV in NSCLC [33,34]. Nevertheless, as in other solid tumor types, a reliable biomarker to identify patients with LADC who will benefit from BEV is yet to be discovered. Here we analyzed the KRAS exon 2 mutational status in a large Caucasian patient cohort (*n* = 501) with stage III–IV, EGFR WT LADC treated with platinum-based chemotherapy alone or in combination with BEV.

In the current LADC cohort, 33.9% of the patients had a KRAS mutation. The incidence of KRAS mutations was higher in the BEV/CHT-treated group as compared to the CHT group (38.5% vs. 29.5%, respectively, *p* = 0.012). With an incidence of 36.8%, G12C was the most frequent subtype in the BEV/CHT group, followed by the G12V (21.1%) and G12D (20%) subtypes. Other rare mutational subtypes (i.e., G12A, G12R, G12S, G13C, and G13D) were identified in 22.1% of the patients. These findings are in line with data previously reported by us and others in large NSCLC studies [12,35].

Next, we investigated whether the KRAS mutational status had an effect on the response to BEV. Although KRAS status had no impact on the OS of LADC patients receiving CHT alone, in the BEV/CHT group patients with a KRAS mutation had a significantly shorter OS. Multivariate analysis also confirmed the role of KRAS as a negative predictor of response to BEV. In lung cancer, so far only two studies have addressed the impact of KRAS mutation on the efficacy of BEV. The results from both of these studies are in line with our data. Chaft et al. treated 50 stage IB–IIIA NSCLC patients in the neoadjuvant setting in combination with CHT and evaluated their pathological response [36]. None of the 10 KRAS-mutant patients responded, in comparison to 11 of 31 KRAS WT cases. Although these authors administered BEV in combination with CHT, based on our current data and also on previous reports from our group and others, the efficacy of CHT is not affected by KRAS status in NSCLC [11,12]. Thus, the better response rate in the KRAS WT group of the Chaft study can be attributed to BEV and not to CHT [29]. In further support of this, Brady et al. studied 93 stage IV NSCLC patients receiving CHT alone or in combination with BEV and observed that, while CHT was as effective in KRAS WT patients as in those with KRAS-mutant tumors, BEV improved OS and PFS in patients with KRAS WT, but not those with KRAS-mutant tumors [30].

Mechanisms of resistance to antiangiogenic agents such as BEV include hypoxia-mediated mechanisms [37], the downregulation of target receptors and the activation of compensatory angiogenic pathways [38,39,40], proangiogenic hematopoietic or endothelial progenitor cell release from the bone marrow [41], inadequate intratumoral distribution of antiangiogenic drugs [42], and a switch from endothelial sprouting to a nonangiogenic vascularization mechanism such as vessel cooption (a frequently occurring vascularization pattern in primary and secondary lung tumors that mediates resistance to anti-angiogenic therapy) [43,44,45,46].) It is not completely clear, though, whether and how KRAS mutation can contribute to these resistance mechanisms. Notably, however, tumor spread through air spaces (STAS) [47] and “tumor islands” [48] are closely related morphological features to vessel cooption [49] and were found to be significantly associated with KRAS mutations in NSCLC [47,48]. Moreover, mutant KRAS has been shown to induce the expression of VEGF in transformed fibroblasts or epithelial cells in vitro. KRAS mutation led to the increased expression of other angiogenic growth factors such as TGF (transforming growth factor)-beta and alpha [50]. Elevated VEGF mRNA levels were detected in tumor cell lines expressing mutant KRAS [51]. Genetic disruption of the mutant KRAS allele in human colon carcinoma cells resulted in decreased VEGF secretion [51]. The transfection of human pancreatic epithelial cells with KRAS12V induced the expression of VEGF and CXC (C-X-C motif) chemokines through Erk and c-Jun signaling and enhanced endothelial tube formation in co-cultures, which could be inhibited by CXC receptor 2 or VEGF targeting [52]. Lastly, doxycycline withdrawal led to tumor regression and endothelial apoptosis in a doxycycline-inducible RAS (rat sarcoma)-driven INK4a (inhibitor of cyclin-dependent kinase 4a) deficient murine model of melanoma [22].

In CRC patients receiving BEV, results on associations between KRAS mutational status and outcome have been inconsistent, with a larger number of studies reporting no associations [23,25,26,53,54] than those demonstrating significant associations [27,28]. Interestingly, however, in a recent CRC study, Fiala et al. demonstrated that G12V and G12A mutation were predictors of shorter PFS and OS, while patients with tumors harboring other KRAS mutations had a similar outcome to those with KRAS WT tumors [27]. Notably, another group reported that the presence of a KRAS G12D mutation was significantly associated with poorer outcome in CRC patients receiving BEV-containing regimens [55]. As for NSCLC, Scheffler et al. recently found that patients with KRAS G12D mutation exhibit a high frequency of co-occurring mutations in the angiogenesis-associated PDGF (platelet-derived growth factor receptor)/PDGF-receptor pathway [56]. In line with this, among the three major codon 12 KRAS mutation subtypes (G12C, G12V, and G12D) G12D proved to be a predictor of poor outcome in our BEV/CHT subcohort. Patients with LADC harboring this mutation had significantly worse PFS and OS than those with tumors harboring other KRAS mutations or WT KRAS.

The biological importance of KRAS mutational subtypes has been demonstrated in a study by Figueras et al., who introduced either a codon 12 or a codon 13 KRAS mutation into NIH3T3 cells and analyzed the VEGF levels and the activity of VEGF promoter in these transfected sublines. Despite the lower VEGF expression, codon 12 mutant tumors exhibited a higher microvessel density, while tumors harboring the codon 13 mutation developed angiogenic sprouts with larger diameters [57].

In our cohort, only two patients carried a codon 13 mutation of KRAS, so we could not evaluate the BEV response in this subgroup. Nevertheless, our study suggests that specific KRAS mutation subtypes can have a major impact on tumor vascularization and, potentially, on the response to anti-angiogenic treatment.

Like all retrospective analyses, our study has limitations. First, although we excluded patients with EGFR-mutant tumors from our study, we did not analyze KRAS-WT patients for additional oncogenic driver mutations. Second, we did not study KRAS-mutant patients for co-occurring mutations in additional tumor-associated pathways [56]. Third, because this large retrospective cohort did not include reliable RECIST (response evaluation criteria in solid tumors) data [58] for all patients, we did not investigate the correlation between KRAS mutational status and tumor response according to RECIST criteria. Finally, because there is a massive body of literature on the predictive and prognostic role of KRAS mutations in CHT-treated LADC patients [9,10,11,12,13,56,58,59,60] and the main aim of the current study was to investigate the relationship between KRAS status and the efficacy of BEV, only the OS but not the PFS data were used in our analyses in the CHT-alone subcohort.

## 4. Materials and Methods

### 4.1. Study Population

In this single-center, retrospective study, 501 consecutive patients with advanced lung adenocarcinoma (LADC) were included and underwent first-line platinum-based (cisplatin or carboplatin) doublet chemotherapy (CHT) with or without BEV at the National Korányi Institute of Pulmonology, Budapest between 2007 and 2016 (Table 1, Figure S1). The addition of BEV to CHT was individually decided by the treating physician in line with the proof-of-concept BEV clinical trials [16,18] and with the EMA summary of BEV characteristics. According to our inclusion criteria, cytologically or histologically verified unresectable stage IIIB or IV LADC patients were included. Patients with uncontrollable hypertension, hypertensive encephalopathy, arterial or grade 4 venous thromboembolism, nephrotic syndrome (grade 4 proteinuria), pulmonary bleeding, gastrointestinal perforation, need for major surgery, or hypersensitivity to BEV were considered not eligible for BEV therapy (Figure S1).

In the BEV/CHT group (*n* = 247), platinum was given together with paclitaxel (84.7%) or gemcitabine (15.3%). In order to rule out the potential confounding effect of different treatment regimens, patients receiving other nonplatinum partners, such as pemetrexed or docetaxel, were excluded from the CHT group (*n* = 254). Additionally, all patients receiving tyrosine-kinase inhibitors in any further line of treatment were excluded. According to the therapy guidelines of the host institute, only ECOG PS 0 or 1 LADC patients were included in this study, since higher PS contradicted the use of cytotoxic chemotherapy. Smoking status, TNM stage, and molecular tumor characteristics (EGFR and KRAS mutational status) were defined at the time of diagnosis. For the calculation of PFS and OS, the date of the first CHT was used. Patients with known EGFR mutations were excluded. Clinical follow-up closed on 1 August 2017. The median follow-up was 21 months in the BEV/CHT group, and 10 months in the CHT group. The study and all treatments were conducted in accordance with the current National Comprehensive Cancer Network guidelines, based on the ethical standards prescribed by the Helsinki Declaration of the World Medical Association and with the approval of the national level ethics committee that included a waiver for this retrospective study (52614-4/2013/EKU). Due to the retrospective study design and the anonymity of the patient records, informed consent was not recommended.

### 4.2. Molecular Diagnosis

All mutational analyses were performed at the time of diagnosis at the 2nd Department of Pathology of the Semmelweis University, as previously described [12,61,62]. DNA isolation was performed from formalin-fixed, paraffin-embedded (FFPE) tissue blocks, cytological specimens of primary tumors, or lymphatic or organ metastases (including pleural effusion).

KRAS exon 2 mutations were identified by microcapillary-based restriction fragment length analysis, as previously described [12,61]. Briefly, a tumor-rich microscopic area on H&E staining had been determined by pathologists prior to macro dissection from FFPE tissue or cytological smears. DNA was extracted using the MasterPure™ DNA Purification Kit (Epicentre Biotechnologies, Madison, WI, USA) according to the instructions of the manufacturer. The microfluid-based restriction fragment detection system was characterized by 5% mutant tumor cell content sensitivity. The density ratio of the mutated band to the WT one was calculated and samples containing >5% of the non-WT band were considered mutation-positive due to the sensitivity threshold. The base-pair substitutions in the mutant samples were verified and determined by sequencing on the ABI 3130 Genetic Analyzer System (Life Technologies, Carlsbad, CA, USA) with the BigDye^®^ Terminator v1.1 Kit.

### 4.3. Statistical Methods

Categorical parameters, such as gender (male vs. female), smoking status (never- vs. ever-smoker), ECOG PS (0 vs. 1), and KRAS mutation status (KRAS-mutant vs. WT) were statistically analyzed by Chi-square test or Fisher’s exact test. Age, as a continuous variable, was analyzed in the different KRAS mutational groups by Mann-Whitney U test as the data were not normally distributed in each group (as per the Shapiro-Wilk normality test). Kaplan-Meier survival curves and two-sided log-rank tests were used for univariate survival analyses. The median follow-up time was calculated by using the reverse Kaplan-Meier approach. The Cox proportional hazards model was used for uni- and multivariate survival analyses to detect the impact of both continuous and categorical factors and to calculate the hazard ratios (HR) and corresponding 95% confidence intervals (CI). For multivariate survival analyses, the Cox regression model was adjusted for age (as a continuous variable), gender (female versus male), smoking (never- vs. ever-smoker), ECOG PS (0 versus 1) and stage (IIIB versus IV). In order to establish potential predictive factors, interaction terms were calculated between KRAS status and other variables (age, sex, smoking status, ECOG PS, and stage) in the adjusted multivariate Cox regression model. *p* values are always two-sided and considered statistically significant below 0.05. Metric data are always shown as median or mean and corresponding range or, in the case of OS and PFS, as median and corresponding 95% CI. All statistical analyses were performed using the PASW Statistics 18.0 package (Predictive Analytics Software, SPSS Inc., Chicago, IL, USA). Graphs were created with GraphPad Prism 8 (GraphPad Software Inc., San Diego, CA, USA).

## 5. Conclusions

In conclusion, when combined with standard first-line chemotherapy, BEV has led to increased OS and thus has been approved in patients with advanced or recurrent nonsquamous NSCLC without targetable molecular abnormalities [16,17,19,20,63,64]. However, although serious efforts have been made to identify patients responsive to BEV, there is as yet no validated predictive biomarker in this field. Here, we present novel evidence for use of BEV in stage III–IV LADC patients with KRAS-mutant tumors- and especially with KRAS G12D-mutant tumors- demonstrating inferior activity of this drug compared to that in LADC patients with non-KRAS-mutant tumors. Our data may not only help to improve the efficacy of BEV, but, through better patient selection, could also help to decrease the unnecessary use of this expensive agent in subgroups of KRAS-mutant human LADC patients.

## Figures and Tables

**Table 1:** Patient characteristics in the bevacizumab/chemotherapy (BEV/CHT) and chemotherapy (CHT) groups.

	No. of Patients (%)		KRAS Status		*p*-Value ^a^
			Wild Type (%)	Mutant (%)	
**A. BEV/CHT**					
All patients	247		152 (61.5%)	95 (38.5%)	
Age (years) ^b^		Median:	62	58	0.09
		SD *:	9.2	8.2	
		Range:	53	44	
Smoking ^c^					
Never-smoker	30 (12%)		24	6	0.008
Ever-smoker	167 (68%)		93	74	
No data (*n* = 50)					
Gender ^c^					
Female	106 (43%)		52	54	0.002
Male	141 (57%)		100	41	
ECOG ^c^					
0	139 (56%)		87	52	0.056
1	108 (44%)		65	43	
Stage ^c^					
III	55 (22%)		38	17	0.16
IV	192 (78%)		114	78	
Survival ^d^					
Median PFS (months)			8.63	7.03	**0.0255**
Median OS (months)			21.57	14.23	**0.0186**
**B. CHT**					
All patients	254		179 (70.5%)	75 (29.5%)	
Age (years) ^b^		Median:	63	61	0.297
		SD *:	7.8	8.7	
		Range:	46	46	
Smoking ^c^					
Never-smoker	21 (8%)		15	6	0.435
Ever-smoker	188 (74%)		135	53	
No data (*n* = 45)					
Gender ^c^					
Female	118 (46.5%)		79	39	0.27
Male	136 (53.5%)		100	36	
ECOG					
0	128 (50.5%)		94	34	0.335
1	126 (49.5%)		85	41	
Stage					
III	66 (26%)		44	22	0.351
IV	188 (74%)		135	53	
Survival ^d,e^					
Median OS (months)			11	10	**0.6771**

^a^*p* value is calculated between wild type and all mutant groups, ^b^ Mann-Whitney test is used in case of continuous variable (age) as the data are not normally distributed (Shapiro-Wilk test), ^c^ Fisher’s exact test is used between categorical variables, ^d^ survival difference between the wild type and the mutant group was calculated using log rank regression analysis, ^e^ PFS was not determined in the CHT group, * SD: standard deviation, BEV/CHT: bevacizumab/chemotherapy, KRAS: V-Ki-ras2 Kirsten rat sarcoma viral oncogene homolog, ECOG: Eastern Cooperative Oncology Group, PFS: progression-free survival, OS: overall survival.

**Table 2:** Clinicopathological variables and progression-free survival (PFS) and overall survival (OS) of lung adenocarcinoma (LADC) patients treated with bevacizumab/chemotherapy (BEV/CHT) in the multivariate Cox proportional hazards model.

Clinicopathological Variables	PFS	OS
**Age (continuous)**		
HR	0.628	0.978
95% CI	0.966–1.021)	(0.955–1.003)
*p*	0.628	0.081
**Gender (female vs. male)**		
HR	0.248	0.390
95% CI	(0.125–0.494)	(0.203–0.751)
*p*	0.001	0.005
**Smoking (never- vs. ever-smokers)**		
HR	0.944	0.968
95% CI	(0.548–1.626)	(0.562–1.669)
*p*	0.835	0.907
**ECOG PS (0 vs. 1)**		
HR	0.765	0.772
95% CI	(0.518–1.129)	(0.523–1.140)
*p*	0.177	0.193
**Stage (III vs. IV)**		
HR	0.879	0.603
95% CI	(0.531–1.455)	(0.365–0.996)
*p*	0.617	0.048
**KRAS status (WT vs. mutant)**		
HR	0.597	0.645
95% CI	(0.402–0.887)	(0.458–0.908)
*p*	0.011	0.012

HR, hazard ratio; CI, confidence interval; ECOG PS, Eastern Cooperative Oncology Group performance status.

## Supplementary Materials

The following are available online at https://www.mdpi.com/2072-6694/11/10/1514/s1. Figure S1. Consort diagram for advanced LADC cases. Consort diagram to demonstrate the selection of stage IIIB/IV LADC cases for BEV/CHT or CHT alone in this study. Where patients were excluded, the reasons for exclusion are indicated. Figure S2. Comparison of survival outcomes in patients with advanced LADC according to treatment regimen. Advanced LADC patients receiving BEV/CHT showed significantly higher median OS compared to those treated with CHT only (median OSs were 24 vs. 10 months, respectively, *p* < 0.0001, log-rank test). Figure S3. Kaplan-Meier curves for the OS of LADC patients treated with CHTalone and LADC patients with KRAS G12D mutations in the BEV/CHT subcohort. Patients with tumors harboring KRAS G12D mutations and treated with BEV/CHT had comparable OS to that of patients with KRAS-WT or KRAS-mutant tumors in the CHT-alone subcohort. Table S1. Correlation of clinicopathologic features, outcome variables and KRAS codon 12 subtypes in patients with advanced LADC treated with BEV (*n* = 95).
