# Supplementary material for: KRAS Mutations Predict Response and Outcome in Advanced Lung Adenocarcinoma Patients Receiving First-Line Bevacizumab and Platinum-Based Chemotherapy
Source: Cancers (Basel). 2019 Oct 9;11(10):1514. doi: 10.3390/cancers11101514 (PMC6827133; doi:10.3390/cancers11101514)
Supplement: Supplementary file 1 [file cancers-11-01514-s001.pdf]

Supplementary Materials: KRAS Mutations Predict Response and Outcome in Advanced Lung Adenocarcinoma Patients Receiving First-Line Bevacizumab and Platinum-Based Chemotherapy

Aron Kristof Ghimessy, Aron Gellert, Erzsebet Schlegl, Balazs Hegedus, Erzsebet Raso, Tamas Barbai, Jozsef Timar, Gyula Ostoros, Zsolt Megyesfalvi, Balazs Gieszer, Judit Moldvay, Ferenc Renyi-Vamos, Zoltan Lohinai, Mir Alireza Hoda, Thomas Klikovits, Walter Klepetko, Viktoria Laszlo and Balazs Dome

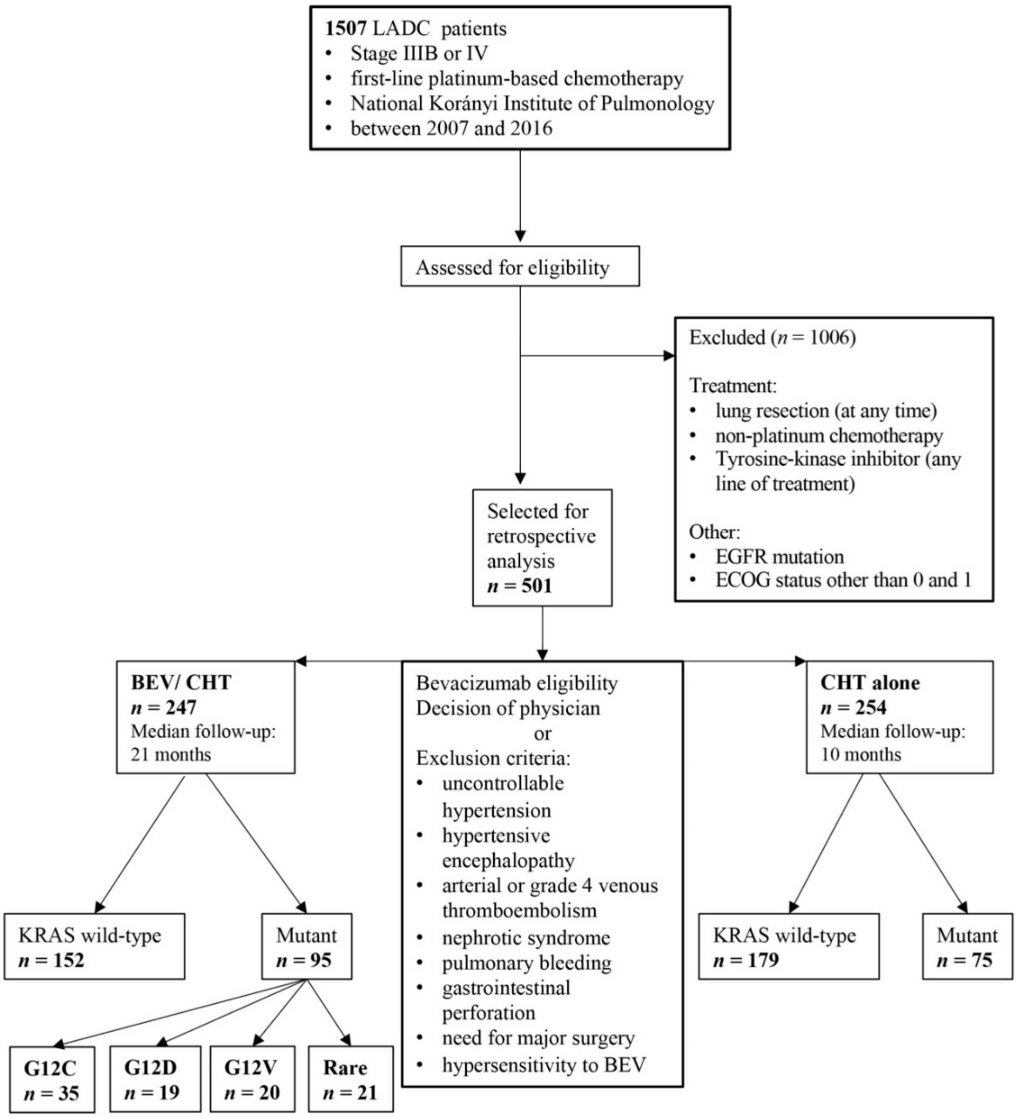

Figure S1. Consort diagram for advanced LADC cases. Consort diagram to demonstrate the selection of stage IIIB/IV LADC cases for BEV/CHT or CHT alone in this study. Where patients were excluded, the reasons for exclusion are indicated.

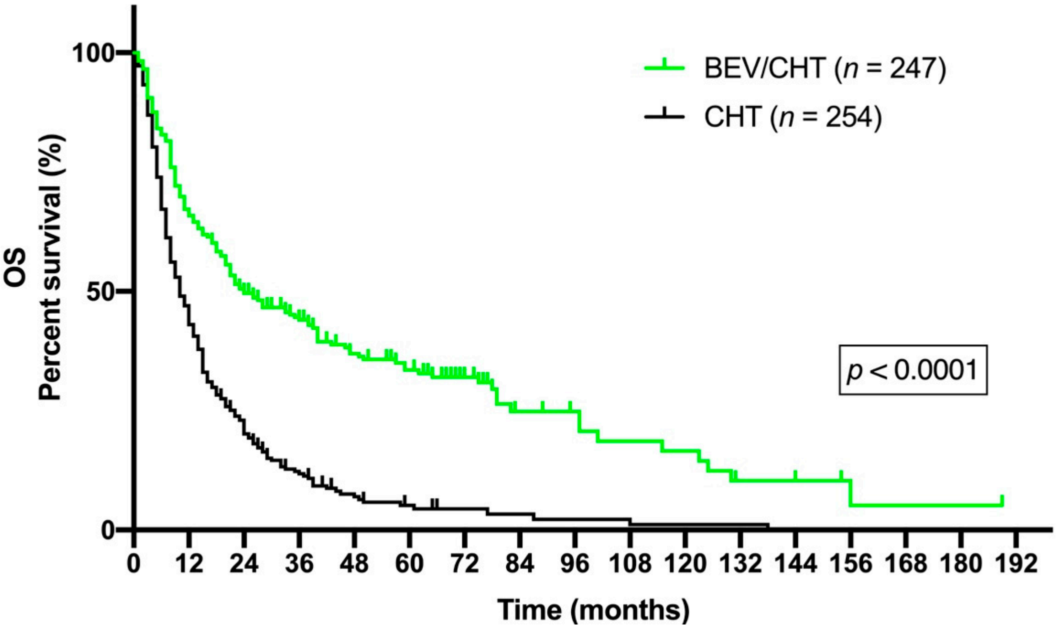

Figure S2. Comparison of survival outcomes in patients with advanced LADC according to treatment regimen. Advanced LADC patients receiving BEV/CHT showed significantly higher median OS compared to those treated with CHT only (median OSs were 24 vs. 10 months, respectively, $p < 0.0001$, log-rank test).

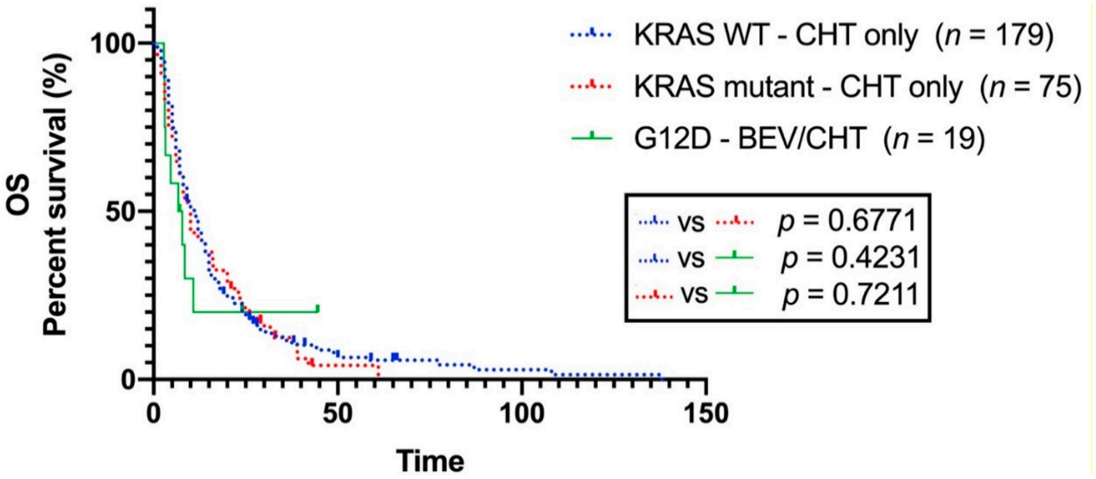

Figure S3. Kaplan-Meier curves for the OS of LADC patients treated with CHT alone and LADC patients with KRAS G12D mutations in the BEV/CHT sub-cohort. Patients with tumors harboring KRAS G12D mutations and treated with BEV/CHT had comparable OS to that of patients with KRAS WT or KRAS mutant tumors in the CHT alone sub-cohort.

Table S1. Correlation of clinicopathologic features, outcome variables and KRAS codon 12 subtypes in patients with advanced LADC treated with BEV ($n = 95$).

All Patients		G12C	G12D	G12V	Rare Mutation	*p*-value[a]
		35	19	20	21	
Age (years)[b]	Median:	61	61	56	56	
	SD*:	7.7	7.1	8.7	7.2	0.579
	Range:	32	30	31	19	
Smoking[c]						
Never-smoker		2	0	2	2	
Ever-smoker		33	15	12	14	0.182
No data (15)						

Gender[c]					
Female	21	6	12	15	0.072
Male	14	13	8	6	
ECOG[c]					
0	17	7	11	17	0.594
1	18	12	9	4	
Stage[c]					
III	12	3	1	1	0.071
IV	23	16	19	20	
Survival[d]					
Median PFS (months)	8,53	3,77	8,17	6.77	0,0057
Median OS (months)	19,20	7,27	14,97	12.97	0.0414

[a] *p* value is calculated between KRAS codon 12 subtypes, [b] Kruskal-Wallis test, [c] Chi-square test, [d] log rank regression analysis, * SD: Standard Deviation.
